# Periscope of Dermatology

**Published:** 1897-11-20

**Authors:** 


### PERISCOPE OF DERMATOLOGY.

### LUPUS.

Morris and Whitfield[1] report the results of the employment of Koch's new tuberculin in six cases of lupus. The new tuberculin is made in the following way: Dry virulent cultures of tubercle bacilli are pulverised in an agate mortar, and the powder so obtained is diffused in distilled water; the fluid is then centrifugalised from the sediment, which latter is dried, powdered, diffused in water, and again centrifugalised; the process being repeated till no sediment forms. The fluid obtained from the first centrifugalisation he calls T.O., and it contains all the toxins of the old tuberculin. The fluids obtained from the subsequent centrifugalisations are those which are now recommended for use, since they contain not the toxins but the components of the bacilli themselves. He calls this fluid T.R.

Following Koch's instructions, they commenced with a small dose ($\frac{1}{500}$ mg.) and raised the dose rapidly; they were careful neither to excite constitutional disturbance nor to give a second dose till the temperature had fallen to normal.

The injection is made beneath the skin between the shoulders or in the side. The following effects of the injection on the lupus were noticed: (1) Diminution of surrounding redness; (2) slight depression in the centre of the nodules, and, later, desquamation; (3) steady healing of all ulcerated surfaces; and (4) slow subsubsidence of all previously permanent œdema. The constitutional disturbance (consisting of fever, headache, and pains in the limbs) was in no case severe, and was always of a transitory nature; no local treatment was employed. The improvement in most of the cases was remarkable, but the authors hesitate to describe the cases as cured, as they are still under treatment.

Morris draws attention to the small supply of tuberculin which is obtainable and to the cost of the treatment, since in some cases each injection has cost 8s. 6d.

Bidwell,[2] after discussing the various methods for the treatment of lupus now in use, including the new tuberculin injections, gives his results of treatment by free excision combined with Thiersch's skin-grafting. Although recurrence occurred in two very extensive cases of long duration, in six other cases no recurrence had occurred after an interval of more than three years. He refers to the good cicatrix left after this treatment, and points out that a true skin with papillæ is developed from the grafts. In this way he accounts for the elasticity of, and the absence of contraction in, the scar.

Popper[3] refers to 34 cases of lupus treated by excision and Thiersch's skin-grafting, in only three of which had there been any recurrence. In some cases the contraction of the cicatrix necessitated a subsequent plastic operation, but, as a whole, the scars were excellent.

Scarenzio[4] reports three cases of lupus which he had treated successfully by hypodermic injections of calomel. In the case of a man, aged 24 years, who was suffering from lupus of the face of seven years' duration, 10 cgr. of calomel were injected into the buttock, and caused rather strong local reaction; five days later there was marked improvement in the sore; after 20 days a second injection of 5 cgr. was given, which was followed by great and permanent improvement. Similar results were obtained in the two other cases reported.

Fournier[5] also reports two cases of lupus which were cured after a few weeks' treatment with hypodermic injections of calomel.

L. Butte[6] has obtained good results in the treatment of lupus by applications of permanganate of potash. A 2 per cent. solution is applied to the part for 10 to 15 minutes every day; this application causes pain for an hour or two, but cocaine ointment speedily relieves this. At the end of a fortnight the nodules are no longer raised, and after a few weeks' treatment in 11 cases the results were all good, and the nodules had entirely disappeared.

Barbe[7] has found that great benefit followed the use of a solution of chlorophenol in alcohol (one in five) in cases of extreme lupus. The solution is applied with a brush every other day. At the end of five months there was marked improvement.

Wild,[8] in a paper on cutaneous tuberculosis, draws attention to the large number of cases of verruca necrogena which are now being reported, and he judges by this that the disease is more common than is supposed. After describing the various tuberculous affections of the skin, he suggests that the variety in the forms of cutaneous tuberculide is due to (1) variations in the virulence of the bacilli; (2) the condition under which the parasite grows, viz., the low temperature of the skin; (3) the character of the soil upon which inoculation takes place (*i e.*, the resisting power of the individual); and (4) the exact depth in the skin at which the inoculation takes place (*i.e.*, whether the bacilli are introduced into the rete malphigi, the dermis, or the subcutaneous tissue). He believes that the great difficulty in curing lupus and other tuberculous lesions is due in many cases to the fact that no precautions are taken to prevent reinfection.

Savill[9] records the case of a man, aged 38, who had 18 years ago developed a patch of lupus verruccosus while employed in leather dressing. The patch was situated on the ulnar side of the back of the left hand, and had slowly increased to the size of two crown pieces. The disease had evidently been contracted from the animals' hides.

Schütz[10] recommends the following new treatment for lupus erythematosus: He paints the affected part with a solution of arsenous acid (1:400·600 or a 4 to 6 times diluted Fowler's solution), twice a day for six days in succession. The swelling and painfulness produced by these applications disappear within eight hours under mild ointments, when the same treatment is repeated. Nine cases treated by this method were cured in an average of eleven weeks. Lupus vulgaris under the same treatment becomes aggravated.

Audry[11] maintains strenuously that lupus is usually, if not always, secondary, and that the primary seat, in the case of the face, is generally in the nasal mucous membrane.

He further states that the tubercle bacillus of Koch has been recently found by Strauss with great frequency on the nasal membrane of individuals apparently in good health. Also the author remarks that most cases of lupoid or verrucose tuberculosis of the limbs are secondary to old, cured, and forgotten lesions of the bones or tendon sheaths.

Leslie Roberts[12] reports a case of lupus erythematosus which invaded the mucous membrane of the lower lip, and states that only one other similar case has been recorded.

Walter Smith[13] describes the case of a girl aged 18 years, who, besides having an undoubted ordinary nodular lupus on the tip of the nose, developed symmetrical exostoses on the nose and fingers, pulpy nodesities on the fingers, and a psoriasis-like eruption on the body and limbs. The tip of the nose was scraped and liquid carbolic acid applied, the scaly patches on the body being treated with white precipitate and carbolic ointments (equal parts). The nose was cured, and the other eruption disappeared under treatment. He considers that all the lesions were tuberculous.

[1] Brit. Med. Jour., July 24, 1897, p. 207. [2] Treatment, Aug. 12, 1897, p. 241. [3] Ditto, ditto, p. 253; Brit. Jour. of Derm., Aug., 1897, p. 339. [4] Gaz. Med. Lombard, April 19, 1897; B. M. J. Ep., July 24, 1897, No. 76. [5] Med. Week, June 4, 1897, p. 262; Brit. Journ. of Derm., Aug., 1897, p. 359. [6] Ann. de la Policlin. de Paris, Jan.; B. M. J. Ep., March 13, 1897 (215). [7] Med. Week, March 19, 1897. [8] Med. Chronicle, March, 1897, p. 416. [9] Brit. Journ. of Derm., June, 1897, p. 241. [10] Chirur. Med. Press, Amer. Med. Surg. Bull. June 10, 1897, p. 254. [11] Journ. des Mal. Cut. et Syph., Dec., 1896; Brit. Journ. of Derm., April, 1897, p. 174. [12] Brit. Journ. of Derm., May, 1897, p. 177. [13] Ditto, ditto, p. 187.

